# “In the hospital, there will be nobody to pamper me”: a qualitative assessment on barriers to facility-based delivery in post-Ebola Sierra Leone

**DOI:** 10.1186/s12978-018-0601-9

**Published:** 2018-09-15

**Authors:** Stefanie Theuring, Alimamy Philip Koroma, Gundel Harms

**Affiliations:** 1 Institute of Tropical Medicine and International Health, Charité- Universitätsmedizin, Augustenburger Platz 1, 13353 Berlin, Germany; 2 Department of Obstetrics and Gynecology, Princess Christian Maternity Hospital, Freetown, Sierra Leone

**Keywords:** Maternal mortality, Facility-based delivery, Post-Ebola, Qualitative study, Sierra Leone

## Abstract

**Background:**

Sierra Leone has one of the highest maternal mortality rates in the world. Encouraging the use of skilled birth attendance in health facilities is an important step in the endeavor to increase the number of safe deliveries. However, public trust in health facilities has been greatly damaged during the Ebola epidemic outbreak in Sierra Leone in 2014/2015, and little is known about external and intrinsic barriers to facility-based delivery (FBD) in the country since the end of the Ebola epidemic.

**Methods:**

We conducted a qualitative study on FBD in Princess Christian Maternity Hospital, Freetown, which is the national referral maternity hospital in Sierra Leone. We performed six focus group discussions with providers, pregnant women and recent mothers surrounding experiences, attitudes and behaviors regarding FBD and potential barriers. Discussions were tape recorded, transcribed and evaluated through content analysis.

**Results:**

Women in our study were overall technically aware of the higher safety linked with FBD, but this often diverged from their individual desire to deliver in a supportive and trusted social and traditional environment. Close relatives and community members seemed to be highly influencial regarding birth practices. Many women associated FBD with negative staff attitudes and an undefined fear. Logistic issues regarding transportation problems or late referral from smaller health centers were identified as frequent barriers to FBD.

**Conclusions:**

More supportive staff attitudes and acceptance of an accompanying person throughout delivery could be promising approaches to increase women’s confidence in FBDs. However, these approaches also imply revising health systems structures, like staff working conditions that are conducive for a friendly atmosphere, sufficient space in delivery wards allowing the women to bring a birth companion, or like the establishment of a reliable peripheral ambulance system to ensure transportation and fast referral.

## Plain English summary

**Background:** Sierra Leone is one of the countries in the world where most women die when giving birth. Assistance of skilled health workers in health facilities increases safety for mothers. However, after many people died in hospitals in Sierra Leone during the Ebola outbreak in 2014/2015, trust in health facilities decreased. Although the Ebola outbreak ended, there might still be barriers to facility-based delivery (FBD).

**Methods:** Our aim was to find out what people think of FBD in Freetown, Sierra Leone. We conducted six group discussions in Princess Christian Maternity Hospital with staff, pregnant women and recent mothers on experiences, attitudes and behaviors regarding FBD and potential barriers.

**Results:** Women in our study were overall aware of FBD being safer than home deliveries. At the same time, they often expressed a desire to deliver in a familiar social and traditional environment. Close relatives and community members often influenced where and how women want to deliver. Many women feared that staff might treat them badly in hospitals and that they might die there. Transportation to hospital from home or from other health centers was named as a problem.

**Conclusions:** Women might be convinced to use FBD if staff would treat them in a more supportive way and if they could bring a trusted person to stay with them during delivery. This means that hospitals need to improve some conditions, like motivating the staff or offering an ambulance system to ensure transportation even from far away.

## Background

Sierra Leone is not only one of the poorest countries in the world; it is also one of the most dangerous countries when it comes to delivering a baby, with a maternal mortality rate of 1360/100.000 births as of 2015. [1] Despite strong efforts to improve health services´ accessibility and availability, e.g. by establishing a peripheral health unit network or reducing financial barriers by introducing the Free Healthcare Initiative for pregnant and lactating women in 2010 [2, 3] immense gaps in the country’s health system in terms of quality of care and patient safety remain a problem. Furthermore, the largely inadequate obstetric care offered by an already weak health system in Sierra Leone has been further compromised during the Ebola epidemic with approximately 10 000 cases between 2014 and 2015, forcing many hospitals to close down or reduce their activity to prevent Ebola transmission or because of staff shortages. [3, 4] Many women in the Ebola outbreak-affected countries including Liberia, Sierra Leone and Guinea are likely to have died due to the reduced access to appropriate care during childbirth, and many more to have lost their trust in the reliability and safety of health services during the virus outbreak [3]. While before 2014, facility-based deliveries (FBD) in Liberia were rising up to an all-time high, after the Ebola epidemic, FBDs decreased by up to almost 80%. [5] In Sierra Leone, before 2014, only about half of deliveries were facility-based [6]. A report assessing changes in health services utilization in the early phase of the Ebola outbreak showed that service uptake was seriously compromised with a one-third decline in facility-based deliveries already by September 2014, mostly as a result of women’s fear and lack of confidence in health services [7, 8]. Since the Ebola outbreak ended, there is uncertainty whether the public trust in health institutions has been restored, and to date, research regarding the ongoing potential intrinsic and extrinsic barriers to FBD is scarce in this post-outbreak population. This study aimed at capturing the current situation regarding attitudes of patients and providers on FBD in Freetown, Sierra Leone.

## Methods

We conducted a cross-sectional qualitative assessment based on six Focus Group Discussions (FGDs) to identify common attitudes, perceptions and behaviors with respect to FBDs, with the goal to design further formative research to increase institutional delivery rates. Our study was located in Princess Christian Maternity Hospital (PCMH) in Freetown, Western Area of Sierra Leone. PCMH is the national referral maternity hospital in the country, with a capacity of about 140 beds and approximately 5700 deliveries in 2016. PCMH is the only facility providing Comprehensive Emergency Obstetric and Neonatal Services in the Western Area, comprising of about 1.5 million inhabitants. [9]

### Study procedures

Within PCMH, we conducted two FGDs with health care providers, two FGDs with pregnant women from the ANC ward, and two FGDs with mothers who were waiting for hospital discharge after delivery. This mixture of groups was supposed to offer insight into a wide range of perspectives from different positions. FGD participants were recruited purposively; recruitment criteria included belonging to one of the three target groups, the person not being involved in any medical procedure at the time of recruitment, and willingness to participate. An information sheet on study participation was read out to all participants, and they gave written informed consent by signature or fingerprint. We recruited five to seven participants for each FGD, based on the assumption that a rather small group size can create an encouraging atmosphere for each individual to engage in the discussion [10]. The number of FGDs was subject to data saturation, which was achieved after six groups as we observed reiterating discussion contents and no new information being brought up within those target groups. A local study nurse moderated the FGDs in English and Krio, using a previously prepared discussion guide. The discussion guide comprised questions surrounding general attitudes towards home delivery, institutional delivery, traditional birth attendants (TBAs), perception of barriers to facility-based deliveries, and own experiences with delivery (patients, as well as providers who had previously given birth) or working at the delivery ward (staff). The discussion sessions were located in a privacy-offering counselling room of PCMH and lasted for about 60 min each, a time seeming appropriate for within-group data saturation. For women who spoke in Krio, the study nurse translated questions to Krio and answers to English immediately. Statements of FGD participants were subject to probing and respondent validation. Discussions were audio-taped; in addition, an observing person took notes. This was supposed to support data interpretation, e.g. to capture group dynamics or for the case of affirmation of a statement through body language. We asked each of the participants to fill in a form for basic demographic information before the discussions started. The participants received a refreshment and a small financial compensation for their time spent. The six FGDs were conducted in October 2017.

### Analysis

We performed a both tape-based and note-based constant comparison analysis of the data, with an abridged, partly (for key statements) verbatim transcription of audiotapes by one researcher. The data went through a three-step process of inductive coding performed by one researcher (the first author), including initial open coding, axial coding, i.e. grouping and linking identified codes within categories, and selective coding, i.e. deriving themes from the identified categories. [10] The coding procedure involved iterative reading of transcripts and notes combined with writing analytic memos to reflect on the emerging concepts. The content analysis was performed manually.

## Results

Thirty-five women took part in our six focus groups; 13 providers and 22 patients. The average age in the provider groups consisting of female nurse/midwives was 43 years, the majority of providers were Christian (9/11; 2 missing data), the minority Muslim (2/11).

Among the patient groups, the average age was 26 years (*n* = 19, 3 missing data). Three quarters of clients were Muslim (15/20, 2 missing data) and one quarter was Christian (5/20). The majority was married (14/17, 5 missing data). Average travel time to the hospital among the patients was 70 min (*n* = 16, 6 missing data). As their occupation, most patients stated they were students or petty traders. Among the participants from the group of recent mothers, the majority had been referred to PCMH for some pregnancy or birth complication.

### General views on FBD

The common understanding among all participants, both patients and providers, was that FBD would generally provide best medical care and least health risk for mother and baby.

*“I think it will be safer for me to deliver in the hospital.”* (Pregnant woman, FGD3)

*“If you come to the hospital, they will know how your baby is, they will know what to do. They will do a scan.”* (Pregnant woman, FGD3)

*“In case of any problem or complication, specialists are available who can help.”* (Pregnant woman, FGD3)

*“I think that is the right way, to deliver in the hospital, because there you can get best medical care.”* (Pregnant woman, FGD4)

*“Home delivery is very risky. (If not going to a health facility), you end up losing the baby and even your own life.”* (Recent mother, FGD6)

*“If you go to the central hospital, and you are delaying and you cannot push, we have a mechanism, the vacuum; we will use it to lift this baby from you. We have so many facilities. (…) If the baby is not responding well, we have a good resuscitation center.”* (Health provider, FGD1)

However, contradicting to the positive attitudes participants were describing, at the same time they expressed a general concept of fear, wariness and mistrust regarding health facilities. Most women reporting FBD experiences mentioned an initial fear of going to hospital for delivery, especially for surgery. Reasons remained opaque, but it was vaguely implied that fear was justified. It appeared as if going to the hospital was widely equated with having to die there.

*“I was frightened, because I never wanted to go to that theater again.”* (Recent mother, FGD6)

*“They told me I am going to need an operation, I feared. (…) I put everything to god.”* (Recent mother, FGD6)

*“Why are women afraid of the hospital? (…) They have their reasons.”* (Recent mother, FGD6)

*“Some women don’t want to be referred to hospital. They think it’s the end of their lives.”* (Recent mother, FGD5)

*“When Ebola was there, women would not come to hospital, because they said if you come, you will be infected, you will die. Because so many people had Ebola. They are afraid of this.”* (Health provider, FGD2)

*“Most of the (women) will always say, when you go to PCMH to deliver, you die.”* (Health provider, FGD1)

*“Some of the women will tell them (smaller health centers), please don’t refer me, I don’t want to go to PCMH, I don’t want to die.”* (Health provider, FGD1)

### Attitudes and behavior of delivery staff

Women who had experienced FBD confirmed predominantly positive experiences, having perceived staff as encouraging and supportive.

*“They took good care of me, they talked to me, advised me and encouraged me.”* (Recent mother, FGD6)

*“I was giving the nurses a hell of a time. (…) The nurse that night, I congratulate her. She went up and down. Four doctors I think she called for me.”* (Recent mother, FGD6)

*“They are gentle (…) I would still come to this hospital”* (Recent mother, FGD5)

*“I like the way they took care of me. Staff encouraged me when I was crying, as I was a (primipara), so they encouraged me a lot.”* (Pregnant woman, FGD3)

*“They only gave the best care to me. They were so close and monitoring everything. They encouraged me so much.”* (Pregnant woman, FGD4)

However, women also reported a number of negative experiences, including communication problems, being neglected by hospital staff, or certain undesirable health workers practices during delivery, like lithotomy position.

*“There was a very long delay. (…) I told (the nurse) to do something. She insisted on what she thinks was best. (…) I was admitted on Monday, I went into labor on Tuesday, but C/S was only done on Saturday. (…) (The baby) was alive when I was admitted, but she was born dead.”* (Recent mother, FGD5)

*“I went to a health center closeby; I was thinking that the nurses were very wicked. They just placed me here, nobody talked to me, nobody looked at me, for about ten hours.”* (Health provider, FGD2)

*“I was in pain, I wanted to go to the toilet. Nobody came to me to help, so I myself went to the toilet, and I felt something was coming out, I called the nurses: `come, look at my baby!` I said, `you people are sitting there, talking, talking, talking, and I delivered my baby by myself.´ They were sitting outside the room; after the examination, they just forgot about me.”* (Health provider, FGD2)

*“Some of the nurses we talk to…there’s so many attitudes when you are in labor. You may have some kind of behavior that the nurses will not like. But they have to be patient because we are in labor. Their boss should tell them to be patient with us.”* (Pregnant woman, FGD3)

*“The majority of us don’t know how to deliver in a bed. We are not used to that. We prefer to deliver on the ground, that is better for us. (…) When you go to the hospital, the nurses will just leave you on the bed. (…) I would like to deliver on the floor. The bed is too high, and we are in a form of fearness.” (Pregnant woman, FGD 3)*

*“All they wanted to do was a vaginal examination. I refused vaginal examination. The area was already sore.” (Health provider, FGD2)*

The problem of negative staff attitudes possibly deterring women from approaching PCMH was particularly expressed by the providers themselves, who argued that a lack of motivation in underpaid and overworked nurses and midwives would substantially contribute to unfavorable working attitudes of staff.

*“It is the attitude of health workers that is not correct. Some of the women will say, ´I don’t want to go to this facility to deliver because the nurses don’t treat me fair, when I go they shout at me.´ (…) The nurses are stressed.”* (Health provider, FGD1)

*“The salary is very low. It is not encouraging. (…) So we are short of staff. This is the reason why most of us will get annoyed with the patients. (…) I perform three deliveries and I am overworked. I’m stressed with my empty pockets. And when you come around with your own problem, you don’t want the problem of others, it just gets me mad.”* (Health provider, FGD1)

*“There is a shortage of health workers. We are dealing with so many patients. So some women may even refuse to come another time, because they say `if I go there, I will stay a long time`. The work is too much for us and there is no motivation. We are working for 24 hours, covering three shifts sometimes. That may lead you to maybe talk a bit hard to the patients.”* (Health provider, FGD2)

Inhibiting staff attitudes also appeared in the context of the consistently mentioned problem of late referral from smaller health centers to hospitals in emergency cases. Hospital staff described health workers of smaller health centers as often ignoring their limits or overestimating their skills, and thereby causing a delay in referring the patient to hospital.

*“The health workers there will be on their limit, but they don’t want the community to see: this health worker doesn’t know her job. (…) If you check our (PCMH) maternal mortality, you will see that most of the (referred) cases come in as `death on arrival´. (…) So now as a midwife or nurse (…) when you see that this particular case, you should not handle it, you should refer earlier.”* (Health provider, FGD1)

*“This (late arrival in hospital) can be the fault of colleagues. Sometimes they hold on to these patients for quite long beyond their limits. By the time they refer the patients, by the time they get here if the distance is too long, they come here already obstructed.”* (Health provider, FGD2)

### Role of trusted person/TBAs

As a logical addition to perceived negative attitudes and communication problems with hospital staff, women repeatedly articulated their desire to have a person of trust around when it comes to delivery, like a relative or a TBA. Since delivering woman are usually not allowed to bring an accompanying person into the delivery ward, they fear being unattended and left alone. This imagination causes discomfort and hesitance to choose FBD in many pregnant women despite their knowledge that it is the safer option.

*“I will be more comfortable to deliver at home. I am afraid that in latency phase, they will admit you and leave you alone. I am afraid to be alone, I want to deliver at home so I can be with the same midwife.”* (Pregnant woman, FGD3)

*“I am not certain where to deliver, I am having fear. I will have someone at home to pamper me, in the hospital, there will be nobody to pamper me.”* (Pregnant woman, FGD3)

*“(Pregnant women) believe in what older women are telling them, giving them information, helping them at home.”* (Pregnant woman, FGD4)

*“People believe they should deliver at home rather than in hospital, because their grandparents have done it, their great-grandparents were doing it, so they prefer to do it.”* (Health provider, FGD1)

The large influence of women’s trusted social environment also caused delay in hospital referral in some cases where an emergency occurred. When asked why they did not approach the hospital immediately when noticing danger signs, women referred to trusted people in their family/community who advised them, often based on wrong concepts of the symptoms.

*“Elder people are experienced. This was my first own pregnancy, so I don´ t have any idea about that. So I took the idea of them. (…) They gave treatments to me.”* (Recent mother, FGD5)

*“I had swollen feet. (My people) said that maybe it is because it is a male child. One day a nurse was passing by. She said: this is a danger sign, go to hospital on Monday. Unfortunately, I was convulsed before Monday. (…) I was having the idea that my feet were swollen because I was having a male child.”* (Recent mother, FGD5)

From the close social environment, especially husbands seemed to be highly influential on the place of delivery. Reports on partner support with respect to FBD were divergent, including both encouraging and inhibiting attitudes. However, participants described women as generally not authorized for such decision-making without the partner and having to wait for his approval, even in emergency cases.

*“My husband also advised me to go to the hospital. Going to the hospital is the best thing.”* (Pregnant woman, FGD4)

*“I asked him why his wife doesn’t deliver in hospital; he told me categorically that whenever his wives, because he has several wives, come to the hospital to deliver, they will advise them on family planning. (…) He believes when you advise his wives (on family planning), they will go out with another man.”* (Health Provider, FGD1)

*“The women don’t have a right to their own opinion. (…) The nurse will tell them ´I have to refer you to another level, where they will continue your treatment`, the woman will say `I want to wait until my husband comes`. (…) The women don’t have a right to make their own decision.”* (Health provider, FGD1)

*“They said she was waiting for the husband. (…) Just as I’m telling you about this knowledge that you should go earlier and save your own life, not your husband’s life, but your own life! She will prefer sitting at home waiting for him.”* (Health provider, FGD1)

TBAs were characterized to have an important role as trusted and respected persons, often with a family bond, whom many women prefer to FBD because of tradition and belief.

*“TBAs are in the community, they are very close to the patients. Sometimes these TBAs are women’s mothers, or mothers-in-law. So they are trusting them more than the nurses in the hospital.”* (Health provider, FGD1)

*“TBAs, the women know them for a long time, so they think they will be handled with care and treated better. (…) Also, some of them are their relatives, so they trust in them more than in nurses.”* (Health provider, FGD2)

*“They are the people that are respected in the community. Most of the nurses, they are just young girls. TBAs, maybe she is my mothers or grandmothers age, so they are most trusted.”* (Health provider, FGD2)

At the same time, participants discussed that TBAs are often lacking medical competencies, and they described the resulting skepticism. Officially, TBAs have no permission to perform deliveries under threat of penalty charge, but recent efforts strived for integrating TBAs into the formal health system. Discussants however agreed that women would never reveal to a health facility if they had been with a TBA, so no one would know or charge a fine.

*“TBAs are not trained to a level to attend to complications”* (Health provider, FGD2)

*“I do not trust them 100% (…) For them, they don’t know how to do. They just say the baby is fine.”* (Pregnant woman, FGD3)

*“I am not comfortable with the TBAs because they don’t know where the baby lays and how to handle complications.”* (Pregnant woman, FGD3)

*“It was decided to let TBAs join our health workers in the hospital setting. (…) They sent out a call to the TBAs: the greater the number of patients you bring to the hospital (for delivery), the higher they pay you. (…) So the TBA will encourage women to come to the hospital.”* (Health provider, FGD2)

### Travel distance/transportation

Participants frequently expressed long travel distance to PCMH as an important obstacle. It was stated that many women have no means of transportation, especially when labor starts during the night. The failure to approach a health facility for delivery was also perceived as a consequence of poverty and the inability to pay for transportation. Oftentimes, TBAs remain the only accessible option of any skilled attendance in the absence of transportation to a health facility.

*“Most pregnant women do not come to the hospital because of transport, because they are living at a distance.”* (Pregnant woman, FGD4)

*“At longer distance, pregnant women will not have transport. Poverty plays a big role to get to the hospital.”* (Pregnant woman, FGD4)

*“Patients go home first to raise money. They are coming here, they don’t know anybody, for transport, for food, they need money to take care of themselves. These are very poor patients. Even the blood transfusions have to be paid.”* (Health provider, FGD2)

*“There are places that are not motorable and are far away from any health center. So most of these people now will prefer to go to the TBA.”* (Health provider, FGD1)

In some cases, PCMH staff had contributed to the transportation barrier to FBD by sending a delivering woman back home because she was not far enough progressed in labor. Staying closer to the hospital around the due date was discussed as an option.

*“I came in labor, but it was the latency phase, they returned me back home. Then later, labor progressed in the middle of the night, so I was unable to come back to the hospital, because I was living out of Freetown. (…) I went to a retired midwife and delivered at home because of the distance.”* (Pregnant woman, FGD3)

*“If I lived far off, I would think about a relative that stays very close to the health facility, so about one or two weeks before my delivery time, I go closer to the health center and stay with my sister who is already there. So by the time of contractions, I don’t need to delay by getting to the health facility (…) It would ease so many problems.”* (Health provider, FGD2)

*“In PCMH, there is a waiting room. But sometimes the patients refuse to stay, because if they have their farm work to do, they will not leave it there, so they refuse to stay. Also this waiting area is only for women with high risk pregnancy, so the others have to go home.”* (Health provider, FGD2)

The participants did not consider the ambulance system as helpful to facilitate transportation because of lack of sufficient ambulance cars. Even if women have a possibility to go to PCMH by car, public transport or ambulance, they mentioned that heavy traffic in Freetown would not permit easy access to the hospital.

*“Ambulance is there, but there is no fuel, the relatives need to provide fuel, and they don’t have the money. Also, the ambulances only cover the district headquarters. They are not in the villages.”* (Health provider, FGD2)

*“That is another problem. Most of the times these (smaller) health centers don’t have an ambulance. So the time they will call for an ambulance, you think of the traffic, you think of the poor road network, the condition of the patient should have gone worse. Most of these patients are dead on arrival”* (Health provider, FGD1)

## Discussion

This qualitative assessment aimed at reflecting patients´ and providers´ current frames of mind regarding institutional delivery in a setting characterized by extreme poverty, high maternal mortality and ongoing recovery from a major health system collapse during the Ebola outbreak. We observed heterogeneous perspectives on FBD, and identified several intrinsic as well as extrinsic barriers, which were largely concurring with outcomes from a recent systematic review of qualitative evidence on facilitators and barriers to FBD in low- and middle income countries [11].

Overall, study participants expressed a general agreement on health facilities being the safest and most reasonable option for delivering a baby. This confirms earlier findings from Tanzania, where women were highly knowledgeable about the fact that complications during delivery are best manageable at health facilities [12]. Bohren et al. likewise endorsed that health facilities were usually perceived as providing high quality care [11].

At the same time, and somewhat contradictory, the strong perception of advantages of FBD stood alongside a frequently expressed fear of hospital admission and preference for home delivery. This discrepancy, largely acting between the poles of rationality and emotionality, represents a major intrinsic conflict in the context of FBD. The underlying scheme is also transferable to what Bohren et al. have described as an “intersection of traditionalism and modernity” [11]. While “modernity” equates to decision-making in favor of high-standard medical care and obstetric technologies based on the rationale that this will result in increased safety and survival, the “traditionalism” aspect is strongly linked to the influence of a woman’s social and cultural environment and the desire to be accompanied and emotionally supported by a trusted person. An event like the Ebola outbreak is likely to increase emotions like fear and mistrust in authorities, and thereby counteracts allegedly rational arguments for institutionalized health care, as also suggested in a systematic review on effects of Ebola on health care utilization [4]. Other studies corroborate the general relevance of our finding that familiar support and care during home delivery might outweigh reasoning for FBD for many women [13, 14].

This is even more the case when women were having bad experiences with inattentive, careless or even rude health staff at the hospital. Unfavorable attitudes of staff were subject to a number of studies in the context of FBD [15–17], and are a constant emerging theme when describing experiences with FBD, ranging from verbal abuse and ignorance to even physical abuse like slapping. A recent study from rural Sierra Leone observed that poor women were particularly prone to unsupportive staff attitudes, with health facilities structurally reflecting social power imbalances within a society [2]. Oyerinde et al. [16] argued that humanized care based on women’s dignity during delivery is one of the major determinants of patient satisfaction with maternity services and vital for the public perception of FBD, while a study in Benin concluded that it could even positively influence the progress of birth, like less need of medication [18]. Several studies confirm our finding that patients and especially providers accuse the health system to be responsible for overloaded staff and unbearable working conditions, resulting in a climate of demotivation, frustration and harshness [15]. In any case, respectful and attentive staff attitudes are crucial when aiming at building confidence among delivering women in an institutional environment where they do not know anyone and are in a state of fear. To compete with the trusted social environment of a woman during home delivery, health service implementers need to recognize the pertinence of this topic, and consequently address unfavorable working conditions of staff as well as increase their social and communicative skills in stressful situations. The option of allowing an accompanying person into delivery wards to support a woman throughout the process of giving birth, as it has been practiced in European countries for several generations, could be a worthwhile consideration to improve FBD attractiveness [15, 19, 20]. Continuous emotional support and advocacy may result in increased feeling of control and competence in the delivering woman, with multiple potential benefits. As a Cochrane trial review on the topic showed, one-to-one birth companionship of any person, including unskilled family members, was not only associated with fewer negative views on delivery of the women, but also with improved birth outcomes, like more spontaneous deliveries and less pain medication. [20] In many resource-poor health settings including PCMH, allowing additional persons´ presence in delivery wards is challenging because of restricted space conditions. However, given the importance of this theme and the simplicity of this no-cost measure, health decision makers should take into serious account how this could be realized and included into health policies.

Long distance to the hospital and lack or unaffordability of transportation was among the strongest extrinsic barriers women mentioned for FBD. Poor geographic access and prohibitive costs of transportation are a key barrier for seeking institutional, particularly emergency obstetric care in the sub-Saharan African Region and have been recognized as such for a long time. [2, 11–13, 21, 22] Yet, to date, little innovative thought has been conceded to the question of how delivering women in these resource-poor and often secluded settings can reach a health facility at the right time [23]. As it stands, ambulance systems are not a reliable nor affordable option in our Sierra Leonean setting as well as in many similar other ones. Recent research on a free ambulance system in Ethiopia has shown that its uptake halved pregnancy-related mortality in the related districts, strongly suggesting that this could be a highly valuable strategy if further pursued [23]. Ways to establish free, continuously and widely available ambulance systems covering remote areas should be included as a priority issue into health strategic plans with the goal of reducing maternal mortality.

### Limitations

This is one of the first studies after the 2014–2015 Ebola outbreak that looks into perspectives on FBD in Sierra Leone. A limitation of our study is the restriction to participant recruitment from only one health facility, PCMH. Further assessments should involve different facility types including private clinics and peripheral health units, and should also be conducted in other parts of the country including non-urban settings, to be able to generalize our results. Also, we cannot entirely exclude response bias given the FGDs were conducted within the hospital setting; however, all participants were assured that nothing they would discuss would be later attributed to them personally, and our findings generally suggest that there was no shyness among participants to express critical opinions. Logistic constraints of the study setting did not allow for conducting FGDs with home-delivering women directly in their communities or with other influential groups including TBAs and elderly people, nor for comparing experiences of PCMH clients to women having delivered in a facility allowing birth companions in the delivery ward. These aspects could have broadened our spectrum of findings, and further research in this regard would certainly be valuable.

## Conclusion

In conclusion, women in our study setting were aware of the higher maternal and newborn safety linked with FBD, but this often diverged from their individual desire to deliver in a supportive and trusted social and traditional environment. In this desire, they seem to be highly susceptible to the influence of close resource persons like husbands or elder family members. Logistic issues with long travel distances and late referral from smaller health centers appeared as another frequent problem for FBDs. Accommodating delivering women’s emotional state of insecurity and fear by more supportive staff attitudes and the possibility of an accompanying person at her side throughout the process of giving birth could be promising approaches to increase confidence in health institutions. However, these approaches also imply revising health systems structures, like working conditions of hospital staff conducive for a friendly atmosphere, like sufficient space in delivery wards allowing the women to bring a birth companion, or like the establishment of a reliable peripheral ambulance system. In Sierra Leone, where more women die during childbirth than in virtually any other country, it should be a primary goal of health policy makers to mitigate the barriers to FBD in every possible way, and further implementation research assessing effectiveness of the different approaches would be highly desirable for the future.
